# Prognostic factors and survival prediction in HER2‐positive breast cancer with bone metastases: A retrospective cohort study

**DOI:** 10.1002/cam4.4326

**Published:** 2021-10-06

**Authors:** Xiaoshuang Lyu, Bin Luo

**Affiliations:** ^1^ Department of General Surgery, Beijing Tsinghua Changgung Hospital, School of Clinical Medicine Tsinghua University Beijing China

**Keywords:** bone metastasis, breast cancer, HER2, nomogram, prognosis, SEER

## Abstract

**Background:**

Bone is the most common metastatic site of breast cancer. The developmental pattern of bone metastasis differs in different molecular subtypes. The prognostic factors of HER2‐positive breast cancer with bone metastases require further investigation. The goal of this retrospective study was to identify the clinical features and prognostic factors for HER2‐positive patients with bone metastases.

**Methods:**

A total of 34,084 HER2‐positive breast cancer cases and 1204 cases of bone metastases from the Surveillance, Epidemiology, and End Results (SEER) database from 2010 to 2015 were analyzed to identify clinical characteristics and prognostic factors. A nomogram was constructed based on the Cox proportional hazards regression model. The C‐index, calibration curve, and receiver operating characteristic (ROC) were utilized for model validation.

**Results:**

In the HER2‐positive breast cancer total population (34,084 cases), 6.2% developed metastatic diseases. Bone metastases accounted for 3.5% of the entire cohort and 56.7% of all metastatic cases. Univariate and multivariate Cox regression analyses identified seven prognostic factors for predicting cancer‐specific survival (CSS) for HER2‐positive breast cancer patients with bone metastases, including age, brain metastases, liver metastases, lung metastases, PR status, surgery, and chemotherapy. The C‐index of the nomogram was 0.74 vs. 0.78 (for 3‐year CSS) and 0.77 vs. 0.81 (for 5‐year CSS) in the model and validation cohorts, respectively. The AUCs were 0.74 vs. 0.78 (for 3‐year CSS) and 0.77 vs. 0.81 (for 5‐year CSS) in the model and validation cohorts, respectively. The calibration curves indicated favorable agreement between the actual observations and the predictions.

**Conclusion:**

Our study provided population‐based clinical features and prognostic factors for HER2‐positive breast cancer patients with bone metastases and we constructed a prognostic nomogram with reliable accuracy.

## Graphical abstract

Our study identified prognostic factors and constructed a prognostic model for HER2‐positive patients with bone metastases. Seven prognostic factors of predicting cancer‐specific survival (CSS) for HER2‐positive breast cancer patients with bone metastases were identified, including age, brain metastases, liver metastases, lung metastases, PR status, surgery, and chemotherapy.

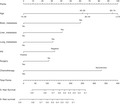

## 1 INTRODUCTION

Breast cancer is the most frequent malignancy and one of the main causes of cancer death in women.^1^ Over the past decades, therapeutic options have been developed, and early breast cancer is now considered curable, with a cure rate of approximately 70%–80%.^2^ However, the main treatment goals for metastatic disease are to prolong survival and control complications, since advanced breast cancer remains incurable using current therapeutic options.^3^ Bone is the most common site for distant metastasis in breast cancer and it constitutes approximately 70% of all metastatic cases.^4^ Bone metastases in breast cancer patients can cause a series of complications, including severe pain, pathologic fractures, hypercalcemia, and spinal cord compression, which bring extreme inconvenience to physical activities and impair quality of life.^5^ Although bone metastasis is regarded as an incurable disease, prolonged survival can be achieved by a combination of systemic therapy and local treatment.^6^

The bone metastatic pattern is highly related to the breast cancer molecular subtypes, with the highest prevalence in the HR‐positive subgroup, followed by the HER2‐positive and triple‐negative subtypes.^7^ Gene expression analysis revealed that over 60% of primary breast cancers with bone metastases are ER‐ and PR‐positive.^8^ However, only 18% of cases with bone metastases are accompanied by HER2 positivity.^9^ The risk factors for bone metastases have been identified in several studies, including age, menopausal status, BMI, grade, tumor size, and lymph node involvement, but the conclusions remain controversial.^10^, ^11^ In addition, several prognostic factors of initial bone metastases have also been identified based on the overall population, including age, marital status, grade, molecular subtype, and surgery condition.^9^

In the HER2‐positive subtype, brain metastasis has been a spotlight of scientific research over past decades due to insufficient delivery of anti‐HER2 drugs through the blood–brain barrier (BBB).^12^ Recently, the small‐molecule tyrosine kinase inhibitor (TKI) tucatinib was proven efficacious in heavily pretreated HER2+ BC patients with brain metastasis, improving both progression‐free survival (PFS) and overall survival (OS) in the HER2CLIMB trial.^13^ Bone metastasis, however, has not been fully explored. A recent preclinical study revealed the molecular mechanism of the association between HER2 and bone metastasis. HER2 activation by knockdown of AKT3 in a bone‐seeking MDA‐MB‐231 cell line promotes metastasis to bone in vivo.^14^ Although limited, these studies indicate that HER2‐positive breast cancer individuals with bone metastases might exhibit distinct characteristics in terms of survival and the response to therapy.

Compared with HER2‐negative patients, HER2‐positive individuals are less likely to develop bone metastases.^15^ Due to its low prevalence, the limited number of cases of HER2‐positive breast cancer with bone metastases has led to difficulty in analyzing the prognosis in this subgroup.^16^ To date, there have been no studies that discussed whether the HER2‐positive breast cancer subgroup represents a unique risk factor pattern for bone metastases. The Surveillance, Epidemiology and End Results (SEER) database, which includes 18 registries that cover 30% of the US population, can provide sufficient data on HER2‐positive breast cancer with bone metastases. Our study analyzed the prognostic factors of HER2‐positive breast cancer with bone metastases and constructed a prognostic model and a nomogram based on the SEER database. Furthermore, we provided estimated survival time probabilities for this small group of patients.

## 2 METHODS

### 2.1 Study population

We obtained HER2‐positive breast cancer data from the SEER 18 Regs Custom Data (with additional treatment fields), Nov 2018 subincidence (1975–2016 varying) database through SEER*STAT 8.3.6. Patients with the following criteria were included: (1) patient data from 2010 to 2015; (2) primary breast cancer; (3) positive HER2 status; (4) complete survival months available; and (5) cancer‐specific survival (CSS): alive or dead due to cancer. Patients with the following criteria were excluded: (1) diagnostic confirmation (the best method used to confirm the presence of the cancer being reported during the entire course of the disease): clinical diagnosis only, unknown whether microscopically confirmed or death certificate only; (2) type of reporting source: autopsy only or death certificate only; and 3) unknown survival months.

The following variables were included in the analysis: age, race, marital status, grade, TNM stage (AJCC 7th edition), brain metastases, liver metastases, lung metastases, number of tumors, surgery, chemotherapy, radiation therapy, ER status, PR status, survival months, and vital status. A total of 34,084 cases were included as the HER2‐positive breast cancer population. Finally, a total of 1204 cases with bone metastases were included in the Cox regression prognostic model, with 858 (70%) in the model construction group and 346 (30%) in the validation group by random distribution using SPSS software.

### 2.2 Statistical analysis

Univariate Cox proportional hazards regression analysis was performed to identify variables that correlated with CSS to a significant extent (*p *< 0.05). Univariate variables with *p *< 0.05 were included in the least absolute shrinkage and selection operator (LASSO) analysis for verification of overfitting. Then, multivariate Cox proportional hazards regression analysis was performed to construct the prognostic model of CSS based on the variables screened above in a Wald backward stepwise regression method. Based on the Cox regression model, we constructed a nomogram for the prediction of 3‐year and 5‐year CSS rates in HER2‐positive breast cancer with bone metastases.

The nomogram was validated in both the model training cohort and the validation cohort. To evaluate the discriminative ability, we constructed a receiver operating characteristic (ROC) curve. The model discrimination power was assessed by the C‐index and the area under the curve (AUC). The C‐index and AUC basically range from 0.5 to 1, with more accurate predictions when they are closer to 1. Generally, values over 0.7 indicate a good model with moderate prediction power.^17^ An ROC curve also contains more information about accuracy, sensitivity, and specificity.^18^ Calibration curves were constructed using a bootstrap method for 1000 resamples to compare the compatibility of the predicted and realistic CSS. Furthermore, patients were divided into low‐risk and high‐risk groups based on the median risk score of the nomograms in both the training and validation cohorts. CSS between the two groups was compared by Kaplan–Meier curves and log‐rank tests.

All statistical analyses were conducted by using SPSS (version 25.0; SPSS, Chicago, IL) and R software (version 3.6.3; http://www.R‐project.org/).

## 3 RESULTS

### 3.1 Patient characteristics

A total of 34,084 patients diagnosed with HER2‐positive breast cancer from 2010 to 2015 were included in this study. Among these, a total of 1204 patients were defined as the bone metastatic subgroup. In the HER2‐positive population, 6.2% had distant metastases, and bone metastases accounted for 3.5% and 56.7% of the total population and the metastatic cases, respectively. Compared with the HER2‐positive breast cancer populations, patients with bone metastases had a higher percentage of solitude (50.7% vs. 39.3%), higher T and N staging, and more additional metastatic sites, especially the liver (37.4% vs. 2.4%) and lung (27.2% vs. 2.0%) (Table 1). For treatment choices, the bone metastasis subgroup tended to receive chemotherapy (79.2% vs. 76.4%) and radiation therapy (41.2% vs. 49.8%) rather than surgery (35.3% vs. 92.0%) (Table 1).

**TABLE 1:** Patient characteristics in the study

Characteristics	Total HER2‐positive population *N* = 34,084 (%)	Total patients with bone metastases *N* = 1204 (%)	Model cohort *n* = 858 (%)	Validation cohort *n* = 346 (%)
Age				
15–29	418 (1.2)	30 (2.5)	20 (2.3)	10 (2.9)
30–44	5808 (17.0)	221 (18.4)	148 (17.2)	73 (21.1)
45–59	14,303 (42.0)	502 (41.7)	367 (42.8)	135 (39.0)
60–74	10,359 (30.4)	334 (27.7)	241 (28.1)	93 (26.9)
75+	3196 (9.4)	117 (9.7)	82 (9.6)	35 (10.1)
Race				
American Indian/Alaska Native/Asian/Pacific Islander	4098 (12.0)	103 (8.5)	68 (7.9)	35 (10.1)
Black	4324 (12.7)	207 (17.2)	147 (17.1)	60 (17.3)
White	25,662 (75.3)	894 (74.3)	643 (74.9)	251 (72.5)
Marital status^a^				
Solitude	13,395 (39.3)	611 (50.7)	430 (50.1)	181 (52.3)
Cohabitation	20,689 (60.7)	593 (49.3)	428 (49.9)	165 (47.7)
Grade				
I	1704 (5.0)	21 (1.7)	18 (2.1)	3 (0.9)
II	12,260 (36.0)	462 (38.4)	323 (37.6)	139 (40.2)
III	19,948 (58.5)	719 (59.7)	515 (60.0)	204 (59.0)
IV	172 (0.5)	2 (0.2)	2 (0.2)	0 (0)
T stage				
T0–1	16,082 (47.2)	154 (12.8)	114 (13.3)	40 (11.6)
T2	13,043 (38.3)	422 (35.0)	301 (35.1)	121 (35.0)
T3	2976 (8.7)	234 (19.4)	168 (19.6)	66 (19.1)
T4	1983 (5.8)	394 (32.7)	275 (32.1)	119 (34.4)
N stage				
N0	19,554 (57.4)	230 (19.1)	167 (19.5)	63 (18.2)
N1	10,356 (30.4)	609 (50.6)	425 (49.5)	184 (53.2)
N2	2463 (7.2)	161 (13.4)	119 (13.9)	42 (12.1)
N3	1711 (5.0)	204 (16.9)	147 (17.1)	57 (16.5)
M stage				
M0	31,957 (93.8)			
M1	2127 (6.2)			
Bone metastases^b^				
No	32,880 (96.5)			
Yes	1,204 (3.5)			
Brain metastases				
No	33,929 (99.5)	1105 (91.8)	789 (92.0)	316 (91.3)
Yes	155 (0.5)	99 (8.2)	69 (8.0)	30 (8.7)
Liver metastases				
No	33,258 (97.6)	754 (62.6)	540 (62.9)	214 (61.8)
Yes	826 (2.4)	450 (37.4)	318 (37.1)	132 (38.2)
Lung metastases				
No	33,388 (98.0)	877 (72.8)	626 (73.0)	251 (72.5)
Yes	696 (2.0)	327 (27.2)	232 (27.0)	95 (27.5)
Number of tumors				
1	31,931 (93.7)	1130 (93.9)	809 (94.3)	321 (92.8)
>1	2153 (6.3)	74 (6.1)	49 (5.7)	25 (7.2)
Surgery^c^				
No	2743 (8.0)	779 (64.7)	556 (64.8)	223 (64.5)
Yes	31,341 (92.0)	425 (35.3)	302 (35.2)	123 (35.5)
Radiation therapy^d^				
No/Unknown	17,098 (50.2)	708 (58.8)	505 (58.9)	203 (58.7)
Yes	16,986 (49.8)	496 (41.2)	353 (41.1)	143 (41.3)
Chemotherapy				
No/unknown	8042 (23.6)	251 (20.8)	185 (21.6)	66 (19.1)
Yes	26,042 (76.4)	953 (79.2)	673 (78.4)	280 (80.9)
ER				
Negative	10,699 (31.4)	346 (28.7)	255 (29.7)	91 (26.3)
Positive	23,385 (68.6)	858 (71.3)	603 (70.3)	255 (73.7)
PR				
Negative	16,164 (47.4)	602 (50.0)	431 (50.2)	171 (49.4)
Positive	17,920 (52.6)	602 (50.0)	427 (49.8)	175 (50.6)
Vital status				
Alive	31,414 (92.2)	647 (53.7)	462 (53.8)	185 (53.5)
Dead	2670 (7.8)	557 (46.3)	396 (46.2)	161 (46.5)

^a^ Marital status. Solitude, the status of living alone. Cohabitation, the status of living with a partner/partners, such as a spouse, relatives, and other companions.

^b^ Bone metastasis, the status of bone involvement at diagnosis. Liver, lung, and brain metastases also represent the status at diagnosis.

^c^ Surgery, surgery of the primary site.

^d^ Radiation therapy, radiation of the primary site.

### 3.2 Cox proportional hazards regression analysis

A prognostic Cox regression model was constructed based on the bone metastasis subgroup of 1204 patients. The model training and validation cohorts were split randomly into 858 (70%) and 346 (30%) cases, respectively. The characteristics of the patients in both cohorts were similar for each variable (Table 1) to avoid selection bias.

**FIGURE 1:**
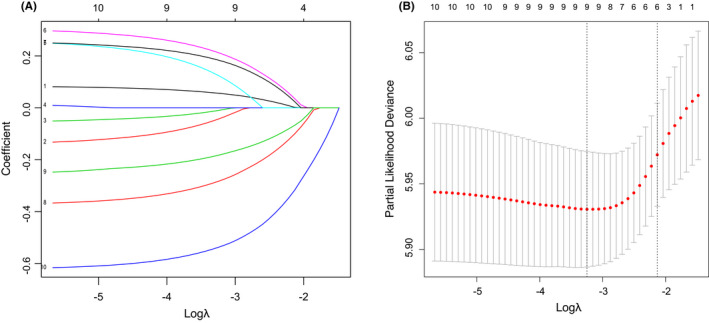
LASSO analysis. (A) LASSO coefficient profiles of 10 variables for CSS. (B) LASSO analysis identified 10 variables for CSS. LASSO, least absolute shrinkage and selection operator; CSS, cancer‐specific survival

**FIGURE 2:**
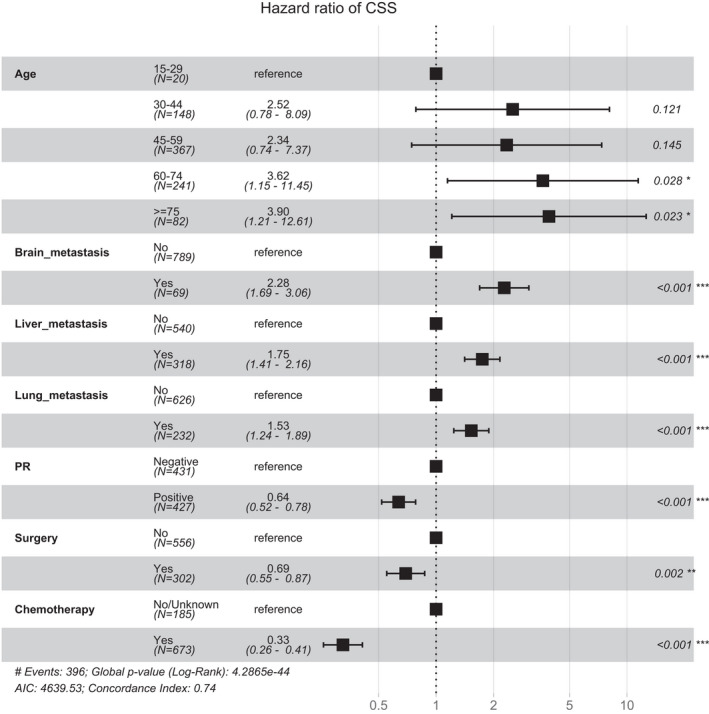
Forest plots visualizing the hazard ratios of the clinicopathologic characteristics for cancer‐specific mortality in the HER2‐positive breast cancer patients with bone metastases

For Cox regression analysis of HER2‐positive breast cancer patients with bone metastases, we identified seven independent prognostic factors in the model training cohort (Table 2). First, we selected 10 variables that were significantly associated (*p *< 0.05) with cancer‐specific survival time based on the univariate analysis, including age, marital status, T stage, N stage, brain, liver and lung metastases, surgery, chemotherapy, and PR status. Lasso Cox regression was performed on these remaining 10 factors, and no variables were excluded (Figure 1). Finally, the 10 factors were included in multivariate Cox proportional hazards regression analyses and excluded in a Wald backward stepwise method. Seven independent prognostic factors for cancer‐specific survival of HER2‐positive breast cancer with bone metastases were identified: age, brain metastases, liver metastases, lung metastases, PR status, surgery, and chemotherapy. Age (HR = 3.63 for 60–74 years), brain metastases (HR = 2.28), liver metastases (HR = 1.75), and lung metastases (HR = 1.53) were adverse prognostic risk factors. PR positivity (HR = 0.64), surgery on the primary tumor (HR = 0.69), and chemotherapy (HR = 0.33) were to be protective factors (Table 2, Figure 2).

**TABLE 2:** Univariate and multivariate Cox regression analyses

Characteristics	Univariate analysis		Multivariate analysis	
	HR(95% CI)	*p* value	HR(95% CI)	*p* value
Age				
15–29	Ref		Ref	
30–44	2.23 (0.70–7.15)	0.177	2.52 (0.78–8.09)	0.121
45–59	3.21 (1.03–10.07)	0.045	2.34 (0.75–7.37)	0.146
60–74	4.42 (1.41–13.90)	0.011	3.63 (1.15–11.45)	0.028
75+	6.26 (1.96–20.01)	0.002	3.90 (1.21–12.60)	0.023
Race				
American Indian/Alaska Native/Asian/Pacific Islander	Ref			
Black	1.13 (0.74–1.71)	0.579		
White	0.88 (0.60–1.28)	0.509		
Marital status				
Solitude	Ref			
Cohabitation	0.79 (0.65–0.97)	0.022		
Grade				
I	Ref			
II	0.77 (0.38–1.56)	0.461		
III	0.86 (0.43–1.74)	0.675		
IV	2.88 (0.61–13.61)	0.181		
T stage				
T0–1	Ref			
T2	0.96 (0.69–1.33)	0.798		
T3	1.10 (0.77–1.58)	0.592		
T4	1.36 (0.99–1.89)	0.061		
N stage				
N0	Ref			
N1	0.72 (0.56–0.92)	0.010		
N2	0.64 (0.45–0.89)	0.009		
N3	0.74 (0.54–1.01)	0.054		
Brain metastases				
No	Ref		Ref	
Yes	2.70 (2.02–3.62)	<0.001	2.28 (1.69–3.06)	<0.001
Liver metastases				
No	Ref		Ref	
Yes	1.71 (1.40–2.08)	<0.001	1.75 (1.41–2.16)	<0.001
Lung metastases				
No	Ref		Ref	
Yes	1.82 (1.48–2.24)	<0.001	1.53 (1.24–1.89)	<0.001
Number of tumors				
1	Ref			
>1	0.98 (0.64–1.49)	0.920		
Surgery				
No	Ref		Ref	
Yes	0.52 (0.41–0.64)	<0.001	0.69 (0.55–0.87)	0.002
Radiation therapy				
No/unknown	Ref			
Yes	1.08 (0.88–1.31)	0.456		
Chemotherapy				
No/unknown	Ref		Ref	
Yes	0.33 (0.27–0.41)	<0.001	0.33 (0.26–0.41)	<0.001
ER				
Negative	Ref			
Positive	0.89 (0.72–1.11)	0.306		
PR				
Negative	Ref		Ref	
Positive	0.71 (0.58–0.87)	0.001	0.64 (0.52–0.78)	<0.001

### 3.3 Nomogram construction

**FIGURE 3:**
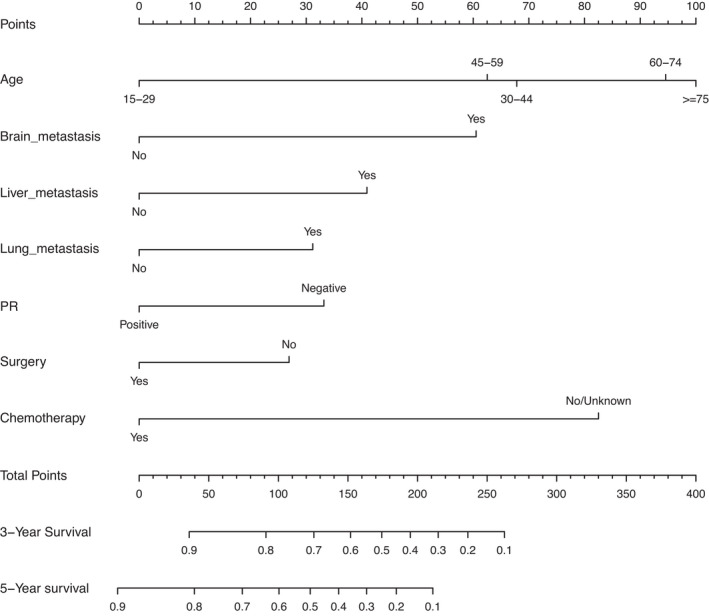
A nomogram predicting CSS in HER2‐positive breast cancer patients with bone metastases. Each prognostic factor was assigned a point on the scale. By adding up all of the points of each prognostic factor, a predicted CSS score can be obtained that corresponds to a certain CSS probability individually. CSS, cancer‐specific survival

Based on a Cox proportional hazards regression model using the previously screened prognostic factors, a nomogram was developed for the prediction of cancer‐specific survival (Figure 3). Each variable was assigned a point scale on the nomogram, representing its contribution to 3‐year or 5‐year CSS. From each point scale, we can intuitively reach the conclusion that age contributed to prognosis the most, with chemotherapy, brain metastases, liver metastases, PR, lung metastases, and surgery closely following. The total points of all seven prognostic factors consisted of the final score for survival, producing the predicted 3‐year and 5‐year survival rates for HER2‐positive breast cancer patients with bone metastases.

### 3.4 Nomogram validation

**FIGURE 4:**
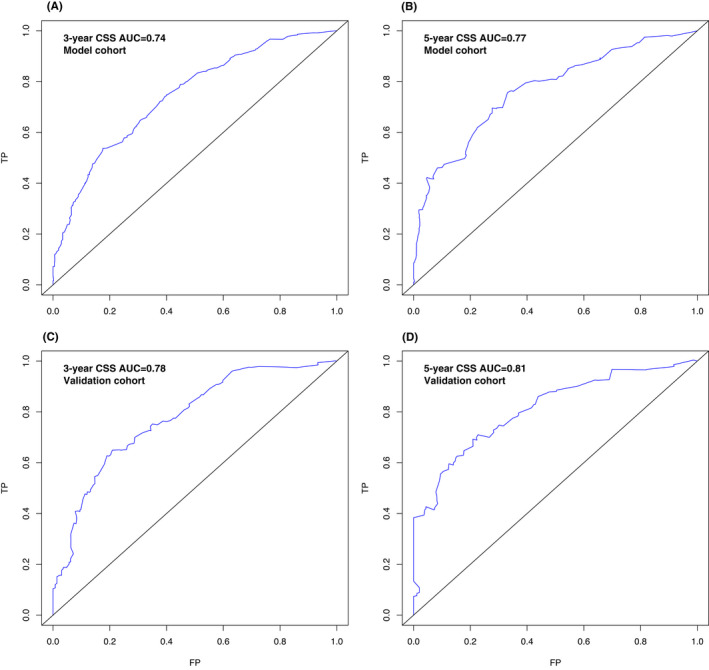
ROC curves of the nomogram for the prediction of 3‐year CSS (A) and 5‐year CSS (B) in the model training cohort. ROC curves of the nomogram for the prediction of 3‐year CSS (C) and 5‐year CSS (D) in the validation cohort. CSS, cancer‐specific survival

**FIGURE 5:**
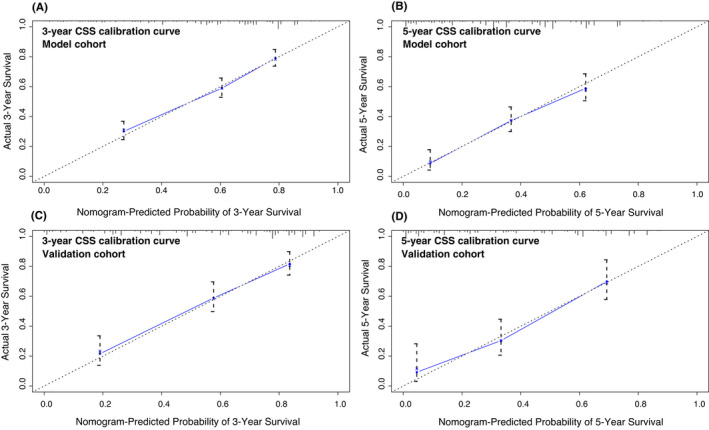
Calibration curves of the nomogram for the prediction of 3‐year CSS (A) and 5‐year CSS (B) in the model cohort (bootstrap = 1000 repetitions). Calibration curves of the nomogram for the prediction of 3‐year CSS (C) and 5‐year CSS (D) in the validation cohort (bootstrap = 1000 repetitions). CSS, cancer‐specific survival

**FIGURE 6:**
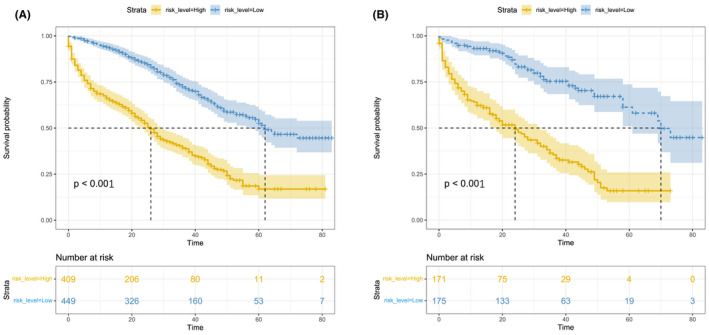
Kaplan–Meier curves of CSS for risk classification based on the risk scores of the Cox regression analysis in the training cohort (A) and the validation cohort (B). CSS, cancer‐specific survival

The nomogram showed medium to strong accuracy for predicting 3‐year and 5‐year cancer‐specific survival, with a C‐index of 0.74 in the model cohort and 0.77 in the validation cohort. Furthermore, the receiver operating characteristic (ROC) curve exhibited similar discrimination power in the model and validation cohorts for both 3‐year (0.74 vs. 0.78) and 5‐year (0.77 vs. 0.81) CSS prediction (Figure 4). The calibration curves for 3‐year and 5‐year CSS both showed favorable accordance in the model and validation cohorts (Figure 5). All cases in the model training and validation cohorts were divided into two subgroups, a low‐risk group and a high‐risk group, according to the cutoff values of the risk scores. Kaplan–Meier survival analysis revealed significantly more favorable CSS in the low‐risk group than in the high‐risk group in both cohorts (Figure 6, *p *< 0.001).

## 4 DISCUSSION

Breast cancer is a heterogeneous malignant disease. Various studies have come to conflicting conclusions about the risk and prognostic factors of bone metastases.^11^ The association of molecular biomarkers, such as HER2, ER, PR, with bone metastases has been identified, with the strongest correlation between bone metastases and HR expression.^19^ Different breast cancer subtypes based on molecular biomarkers exhibit distinct characteristics in terms of tumorigenesis, metastatic patterns, and response to treatment. Hence, it is of great significance to explore clinical characteristics and prognostic factors for each molecular subtype of bone metastases. Our study is the first population‐based, retrospective, prognostic model constructed for this distinct group of HER2‐positive breast cancers with bone metastases. We found that seven clinical features were significantly related to prognosis, including age, brain metastases, liver metastases, lung metastases, PR status, surgery, and chemotherapy.

Interestingly, although T and N stages showed a significant correlation with prognosis in univariate analysis, they had no significance in the multivariate model. The T and N stages, which basically reflect the tumor size and lymph node involvement, reflect the local condition of tumor progression very well but not the status of distant metastasis. A prognostic nomogram study of HER2‐positive early breast cancer demonstrated that T and N stages are powerful prognostic factors for overall survival.^20^ In patients with distant metastasis after surgery, lymph node status and stage have been identified as prognostic factors.^21^ However, in the total population with bone metastases, the results of the prognostic analysis were consistent with our study that T and N stages are not predictive factors of survival.^22^

PR positivity has been identified as a protective factor for the prognosis of HER2‐positive breast cancer with bone metastasis. Previous subtype analyses of breast metastasis also suggested that patients with the HR+/HER2+ subtypes exhibited the most favorable prognosis among all subtypes.^23^, ^24^ Hormone receptor (HR) consists of estrogen (ER) or progesterone receptor (PR), which together constitute the most common luminal subtype and account for 75% of all breast cancer cases.^25^ Our study further investigated the prognostic value of ER or PR status for HER2‐positive breast cancer with bone metastasis and found a significant correlation between PR and prognosis but not for ER. These results narrow down the population of HR+/HER2+ to PR+/HER2+ with the most favorable prognosis of bone metastases.

Other metastatic sites, such as the brain, liver, and lung, had strong effects on the prognosis of HER2‐positive breast cancer with bone metastasis in our analysis. Consistently, studies have proven that patients with bone‐only metastases have prolonged survival and a better prognosis than MBC with multiple bone or additional visceral metastases.^21^, ^26^ Our study further revealed different effects of the metastatic site in cancer‐specific survival. Brain metastases (HR = 2.28) had the predominant contribution to a worse prognosis, followed by liver (HR = 1.75) and lung metastases (HR = 1.53).

Local surgery in the setting of metastatic disease has been controversial in terms of improving the prognosis.^27^, ^28^ However, there has been some evidence supporting its role in improving the prognosis in stage IV breast cancer.^28^, ^29^ In HER2‐positive stage IV breast cancer patients, a retrospective study demonstrated that surgery at the primary site was associated with improved overall survival.^30^ Moreover, the MF07‐01 phase III clinical trial showed significantly improved survival after primary tumor resection with a subsequent ST (systemic therapy) compared with ST only, especially in patients with solitary bone‐only metastases.^28^ Similarly, our study also found that primary site surgery was a protective prognostic factor for HER2‐positive breast cancer individuals with bone metastases. Surgical removal of the primary breast tumor improved both 3‐year and 5‐year cancer‐specific survival among patients developing bone metastases. The therapeutic effect of surgery might act via reducing the tumor burden and enhancing the sensitivity to chemotherapy.^31^

Although behaving as a prognostic factor, surgery had the lowest potency in predicting survival among all of the factors (Figure 2). Notably, bone metastasis data in the SEER database represents the status of bone involvement at the time of diagnosis, which may be the reason why a high percentage of patients (64.7%) did not receive surgery at the primary site. This might explain why the effect of local surgery in a setting of metastatic disease on the prolongation of survival is controversial among different studies.^27^, ^30^ Although local surgery for metastatic breast cancer remains controversial, it might be a therapeutic option for selected patients.

Radiation therapy in our study was considered irrelevant to the prognosis of patients with bone metastasis, which might be explained by the definition of radiation therapy in the SEER database. The radiation code in the SEER database reflects radiation therapy at the primary tumor site, which might explain why it is not a prognostic factor for distant bone metastases.

For HER2‐positive advanced breast cancer, the preferred first‐line regimen should be dual HER2 blockade of trastuzumab and pertuzumab plus chemotherapy based on the CLEOPATRA trial.^32^ For patients previously treated with trastuzumab, the second‐line option T‐DM1 accompanied by trastuzumab and a chemotherapy therapeutic regimen could further improve the overall survival.^33^ The development of small‐molecule tyrosine kinase inhibitors (TKIs), such as lapatinib and neratinib, has also had favorable effects on certain groups of patients.^34^ Our study included chemotherapy in the prognostic nomogram. However, information on anti‐HER2 therapy was lacking due to unavailable specialized treatment data in the SEER database. Although the HER2 expression level of primary tumors and bone metastases varied in several different studies, the main trend indicates HER2 expression is reduced in bone metastatic sites.^35^, ^36^, ^37^, ^38^ In a concordance analysis of 151 cases utilizing bone marrow aspiration, the HER2 status concordance between disseminated tumor cells from bone marrow and primary tumors was only 51%.^36^ However, trastuzumab is still effective in clearing HER2‐positive cells from the bone marrow in patients with bone metastases.^39^ Additional investigations are required to explore the efficacy of various anti‐HER2 agents for bone metastasis patients.

There are several limitations of our study. Due to its use of retrospective cohorts from the SEER database, there was inevitable selection bias and incomplete data in this study. Data related to anti‐HER2 therapy are not available in the SEER database, leaving this significant prognostic factor unexplored. Another limitation is that the metastatic fields in the SEER database only report the presence of metastatic sites at the time of diagnosis. Therefore, the development of metastatic disease during the disease course after initial diagnosis, which is commonly seen in HER2+ BC, was not recorded and explored in our study. In addition, our model validation is based on internal validation via random group splitting. External validation is more convincing than internal validation in terms of accordance and reliability. However, the acquisition of external data is challenging due to the uncommon occurrence of bone metastases in HER2‐positive patients.

In conclusion, our study constructed a prognostic model of HER2‐positive breast cancer patients with bone metastases. We suggest that PR positivity is a protective factor for the prognosis of HER2‐positive breast cancer with bone metastasis. Surgery at the primary tumor site and systemic chemotherapy also prolonged cancer‐specific survival (CSS). Bone‐only metastases had the most favorable prognosis, followed by lung, liver, and finally, brain‐accompanied metastatic sites. The nomogram we constructed provided a reliable and simple method to predict the CSS of HER2‐positive individuals with bone metastasis.

## CONFLICT OF INTEREST

All authors declare no conflict of interest.

## ETHICS APPROVAL

Patient data were obtained from SEER (Surveillance, Epidemiology, and End Results Program), a publicly open database resource. Informed patient consent was not required.

## Data Availability

The original data used to support the findings of this study are available from the SEER (Surveillance, Epidemiology, and End Results Program) database (https://seer.cancer.gov/). The data sets and the R codes used in the current study are available from the corresponding author upon reasonable request.
